# Multidrug resistance transporters P-gp and BCRP limit the efficacy of ATR inhibitor ceralasertib in cancer cells

**DOI:** 10.3389/fphar.2024.1400699

**Published:** 2024-05-02

**Authors:** Xuan-Yu Chen, Zhuo-Xun Wu, Jing-Quan Wang, Qiu-Xu Teng, Hailin Tang, Qianwen Liu, Zhe-Sheng Chen, Wenkuan Chen

**Affiliations:** ^1^ Institute for Biotechnology, St. John’s University, Queens, NY, United States; ^2^ Department of Pharmaceutical Sciences, College of Pharmacy and Health Sciences, St. John’s University, Queens, NY, United States; ^3^ State Key Laboratory of Oncology in South China, Guangdong Provincial Clinical Research Center for Cancer, Sun Yat-Sen University Cancer Center, Guangzhou, China

**Keywords:** multidrug resistance, ATP-binding cassette transporters, P-glycoprotein, BCRP, ATR inhibitor, AZD6738, ceralasertib

## Abstract

The therapeutic effect of chemotherapy and targeted therapy are known to be limited by drug resistance. Substantial evidence has shown that ATP-binding cassette (ABC) transporters P-gp and BCRP are significant contributors to multidrug resistance (MDR) in cancer cells. In this study, we demonstrated that a clinical-staged ATR inhibitor ceralasertib is susceptible to P-gp and BCRP-mediated MDR. The drug resistant cancer cells were less sensitive to ceralasertib compared to the parental cells. Moreover, ceralasertib resistance can be reversed by inhibiting the drug efflux activity of P-gp and BCRP. Interestingly, ceralasertib was able to downregulate the level of P-gp but not BCRP, suggesting a potential regulation between ATR signaling and P-gp expression. Furthermore, computational docking analysis predicted high affinities between ceralasertib and the drug-binding sites of P-gp and BCRP. In summary, overexpression of P-gp and BCRP are sufficient to confer cancer cells resistance to ceralasertib, underscoring their role as biomarkers for therapeutic efficacy.

## 1 Introduction

The Ataxia telangiectasia and Rad3-related protein (ATR) is a serine/threonine protein kinase within the family of phosphatidylinositol 3-kinase-related kinases (PIKKs) (Weber and Ryan, 2015; Menolfi et al., 2023). Other significant members of this protein kinase family include DNA-dependent protein kinase (DNA-PK), Ataxia telangiectasia mutated protein (ATM), and mammalian target of rapamycin (mTOR). These kinases play a crucial role in mediating DNA damage response (DDR), cell proliferation and metabolism (Shaik and Kirubakaran, 2020). In recent years, DDR has become an attractive target in anticancer drug development. Studies confirmed that deficiency in DDR mechanisms are linked to tumor development as many malignant tumors show functional loss or dysregulation of DDR (Goldstein and Kastan, 2015; Lei et al., 2024). Since ATR and ATM kinases are the two key mediators of DDR, inhibitors against these two kinases are under preclinical development and clinical evaluation. ATR kinase is involved in controlling checkpoints in the cell cycle and managing the response to DNA damage resulting from stress associated with DNA replication. Upon activation, ATR phosphorylates multiple substrates to stabilize replication fork, regulate G2–M cell-cycle arrest, the trigger of a delay in the cell cycle allowing more time for the cell to repair the DNA damage or, in the case of failure to do so, initiating programmed cell death in order to prevent inheritance of the damaged DNA (Nam and Cortez, 2011). Hence, inhibiting DDR by targeting ATR has been proposed as a new strategy for cancer therapy, especially in cancer types that have genomic instability due to high replication stress levels (Lecona and Fernandez-Capetillo, 2018). When ATR activity is inhibited, large amounts of single strand DNA accumulate in the genome, leading to substantial fork collapse and cell death, known as replication catastrophe. Without a functional checkpoint by ATR inhibition, cells prematurely enter mitosis with increased DNA damage, causing a mitotic catastrophe (Yano and Shiotani, 2023). Indeed, ATR inhibition has shown promising antitumor efficacy in preclinical models and several ATR inhibitors (ATRi) have entered early phase clinical trials for different cancer subtypes. Ceralasertib (AZD6738) is an oral and selective ATRi developed by AstraZeneca and is currently in clinical phase 1 and 2 trials as monotherapy or in combination with other anticancer agents (Foote et al., 2018; Yap et al., 2021). Recently, two clinical studies reported the promising antitumor efficacy of ceralasertib in combination with the PD-L1 inhibitor durvalumab in advanced gastric cancer (Kwon et al., 2022) and advanced/metastatic melanoma (Kim et al., 2022). It is also in clinical evaluation in combination with PARP inhibitors and chemotherapy for solid tumors (Yap et al., 2021). Although ATRi demonstrates an encouraging anticancer effect, as with other anticancer therapeutics, drug resistance may occur and result in suboptimal response or treatment failure.

Currently, the mechanisms of ATRi resistance are not fully elucidated. Lloyd et al. (2021) utilized the CRISPR-Cas9 genome-wide screening to identify factors that regulate ATRi efficacy. They found that depletion of Cyclin C or CDK8 contributes to ATRi resistance by limiting ATRi-induced replication stress. Another study by O’Leary et al. (2022) reported that loss of UPF2, regulator of nonsense transcripts 2, led to ATRi resistance in multiple gastric cancer cell lines. The UPF2-depleted cancer cells failed to accumulate in G1 phase and circumvented transcription-replication collisions after ATRi treatment. He et al. (2016) reported that Ect2, a Rho GTPase exchange factor, mediates DNA damage response and may contribute to ATRi resistance. Notably, the role of ATP-binding cassette (ABC) transporters P-glycoprotein (P-gp)/ABCB1 and breast cancer resistance protein (BCRP)/ABCG2 in ATRi resistance has not been investigated in previous studies. Since these transporters are major contributors to multidrug resistance (MDR) in cancer therapeutics, it is intriguing to evaluate the role of these MDR transporters in ATRi resistance (Toyoda et al., 2019; Ahmed et al., 2022). MDR is a phenomenon that the cancer cells develop drug resistance phenotype to anticancer agents that have different mechanisms of action and structures (Krishna and Mayer, 2000). Both P-gp and BCRP are able to pump out a variety of chemically dissimilar compounds from the cells, which not only protect the healthy cells from toxins but also render cancer cells MDR phenotype (Goebel et al., 2021). It is well-established that some tyrosine kinase inhibitors (TKIs) can interact with P-gp/BCRP and can be classified as functional modulators or transported substrates. On the one hand, studies showed that tepotinib (Wu et al., 2022), poziotinib (Zhang et al., 2020) and lazertinib (Fan et al., 2022) are able to inhibit the function of P-gp and BCRP in cancer cells, thus mitigating MDR in combination treatment. On the other hand, the pharmacokinetics of frontline TKIs, for example, imatinib (Park et al., 2021) and gefitinib (Agarwal et al., 2010), are affected by P-gp/BCRP. These findings highlight the importance of determining the interaction between anticancer agents and MDR transporters, which could be useful in predicting the drug fate in MDR tumors.

In this study, we explored whether overexpression of P-gp or BCRP in cancer cells can lead to ceralasertib resistance. Furthermore, the combinations of ceralasertib with P-gp and BCRP modulators were evaluated to determine if they can sensitize MDR cancer cells to ceralasertib.

## 2 Materials and methods

### 2.1 Reagents

Colchicine, doxorubicin, mitoxantrone, topotecan, and verapamil were acquired from Sigma-Aldrich (St. Louis, MO). Ko143 was obtained from Enzo Life Sciences (Farmingdale, NY). Ceralasertib (AZD6738) was purchased from MedChemExpress (Monmouth Junction, NJ). The compounds were dissolved in DMSO to create a 10 mM stock solution. Radioactive [^3^H]-vincristine and [^3^H]-mitoxantrone were acquired from Moravek Biochemicals Inc. (Brea, CA).

### 2.2 Cell lines and cell culture

Cancer cell lines and Human Embryonic Kidney (HEK293) cells were grown or cultured in DMEM supplemented with 10% of (v/v) FBS and 1% (v/v) of Penicillin/Streptomycin in a humidified incubator containing 5% CO_2_ and 95% O_2_ at 37°C. KB-C2 originated from the KB-3-1 human epidermoid carcinoma cell line through the application of colchicine-based selection (Lyall et al., 1987). SW620/Ad300 was established from human colon cancer cell line SW620 with doxorubicin selection (Lai et al., 1991). The *ABCB1* gene knockout SW620-ABCB1ko and SW620/Ad300-ABCB1ko cells were previously generated by CRISPR-Cas9 system (Lei et al., 2021). S1-M1-80 was derived from human colon cancer cell line S1 with mitoxantrone selection (Litman et al., 2000). NCI-H460/TPT10 was derived from NCI-H460, human non-small cell lung cancer cell line, with topotecan selection (Lei et al., 2020). The ABCG2 gene knockout NCI-H460-ABCG2ko and NCI-H460/TPT10-ABCG2ko cells were previously generated by CRISPR-Cas9 system (Lei et al., 2020). The HEK293/pcDNA3.1, HEK293/ABCB1, and HEK293/ABCG2 cell lines were kindly provided by Dr. Susan E. Bates (Columbia University, New York, NY) and Dr. Robert W. Robey [National Institutes of Health, Bethesda, MD]. Specifically, the pcDNA3.1 empty vector, the pcDNA3.1 vector containing the full-length gene encoding ABCB1, and the pcDNA3.1 vector incorporating the complete gene sequence encoding wild-type ABCG2 were utilized in the transfection process (Fung et al., 2014). The drug resistant cancer cells were cultured and sustained in adherence to the instructions provided by the supplier.

### 2.3 MTT assay

The IC_50_ values of anticancer drugs were determined by MTT cell viability assay. Cells were plated at a density of 5,000 cells per well in a 96-well plate format. After overnight culture, the drug combinations were added into the designated wells followed by 72 h incubation. To quantify the cell viability, MTT solution (Thermo Fisher Scientific, Waltham, MA) was added into the plates and incubated for 4 h at 37°C. The organic solvent DMSO was employed as the solvent for the resulted formazan crystals and the OD_570_ value was read.

### 2.4 Western blot

Total proteins from ceralasertib-treated cell were extracted using RIPA cell lysis buffer with 1% protease inhibitor cocktail (Sigma-Aldrich, St. Louis, MO). The protein concentration was quantified by BCA assay kit and subjected to SDS-PAGE using the precast polyacrylamide gel (Bio-Rad, Hercules, CA) and transferred onto PVDF membrane (Millipore, Burlington, MA). Membranes were blocked with 5% non-fat milk then probed with corresponding primary antibodies overnight at 4°C. The primary and secondary antibodies were purchased from Thermo Fisher Scientific (Waltham, MA), including P-gp (MA1-26528), BCRP (MAB4155), or GAPDH (MA5-15738). The membranes underwent additional incubation with an HRP-linked secondary antibody (31430), and the resulting protein bands were visualized using an enhanced chemiluminescence (ECL) kit (Thermo Fisher Scientific, Waltham, MA) and ImageJ (NIH, MD) was used to analyze the gray value of each protein band. The protein expression was normalized to loading control GAPDH before statistical analysis.

### 2.5 Immunofluorescence staining

The cellular localization of P-gp and BCRP were visualized using immunofluorescence staining (Wang et al., 2020). Briefly, the cells were plated at a density of 10,000 cells per well in a 24-well plate format. After cell attachment, different conditions were applied to the designated wells. After 72 h treatment, cells were fixed, permeabilized, and blocked with 5% bovine serum albumin (BSA). Antibodies against P-gp (MA1-26528, Thermo Fisher Scientific) or BCRP (MAB4155, Thermo Fisher Scientific) were incubated with the cells overnight at 4°C. The cells were then incubated with Alexa Fluor 488 conjugated IgG secondary antibody (A-11001, Thermo Fisher Scientific, Waltham, MA) and further counterstained with DAPI. Immunofluorescence images were captured by a fluorescence microscope.

### 2.6 [^3^H]-substrate accumulation assay

[^3^H]-vincristine and mitoxantrone were used to evaluate the efflux activity of P-gp and BCRP, respectively (Zhang et al., 2020). Cells were plated at a density of 100,000 cells per well in a 24-well plate format. After overnight culture, the drug combinations were added into the designated wells followed by 2 h incubation. Verapamil and Ko143 were used as reference inhibitors to inhibit the function of P-gp and BCRP, respectively. For the data acquisition, a Packard Tri-Carb Model 1900CA liquid scintillation counter was used (Packard Instrument, Downers Grove, IL).

### 2.7 Evaluation of ATPase activity

Measurement of ATPase activity was performed with P-gp and BCRP crude membrane protein isolated from High Five insect cells using the Hi5-PREDEASY-ATPase kit (SOLVO Biotechnology, Boston, MA) according to the vendor’s protocol with modifications (Chen et al., 2021). The ABCG2 ATPase activity was evaluated using the Hi5-PREDEASY-ATPase kit (SOLVO Biotechnology, Boston, MA) according to the vendor’s protocol with modifications. Ceralasertib (0–80 μM), with or without the ATPase inhibitor Na3VO4–, was incubated with the P-gp or BCRP membrane suspension. The mixtures were incubated at 37°C for 5 min, followed by the addition of 5 mM Mg^2+^ATP to initiate the reaction. The inorganic phosphate released during the reaction period was colorimetrically determined by spectrophotometry. The difference of inorganic phosphate level between groups was used to calculate the quantity of hydrolyzed ATP.

### 2.8 Molecular docking of ceralasertib with human P-gp and BCRP models

The spatial structure ceralasertib was constructed for docking simulation as previously described (Wu et al., 2022). Human P-gp protein model 6QEX (paclitaxel bound) (Alam et al., 2019) and BCRP protein model 6VXI (mitoxantrone bound) (Jackson et al., 2018) were obtained from RCSB Protein Data Bank. Both models are inward facing. Docking calculations were performed in AutoDock Vina (version 1.1.2) (Trott and Olson, 2010). Hydrogen atoms and partial charges were added using AutoDockTools (ADT, version 1.5.4). Docking grid center coordinates were determined from the bound ligands provided in PDB files. Receptor/ligand preparation and docking simulation were performed using default settings. The top-scoring pose (based on affinity score: kcal/mol) was chosen for further analysis and visualization.

### 2.9 Data analysis

All experiments were repeated three times, and the analyzed data was expressed as mean ± SD. Statistical analysis was performed using One-way ANOVA in GraphPad Prism 8.1. A value of *p* < 0.05 was considered statistically significant.

## 3 Results

### 3.1 P-gp and BCRP limited the cytotoxicity of ceralasertib in cancer cells and gene-transfected cells

**FIGURE 1:**
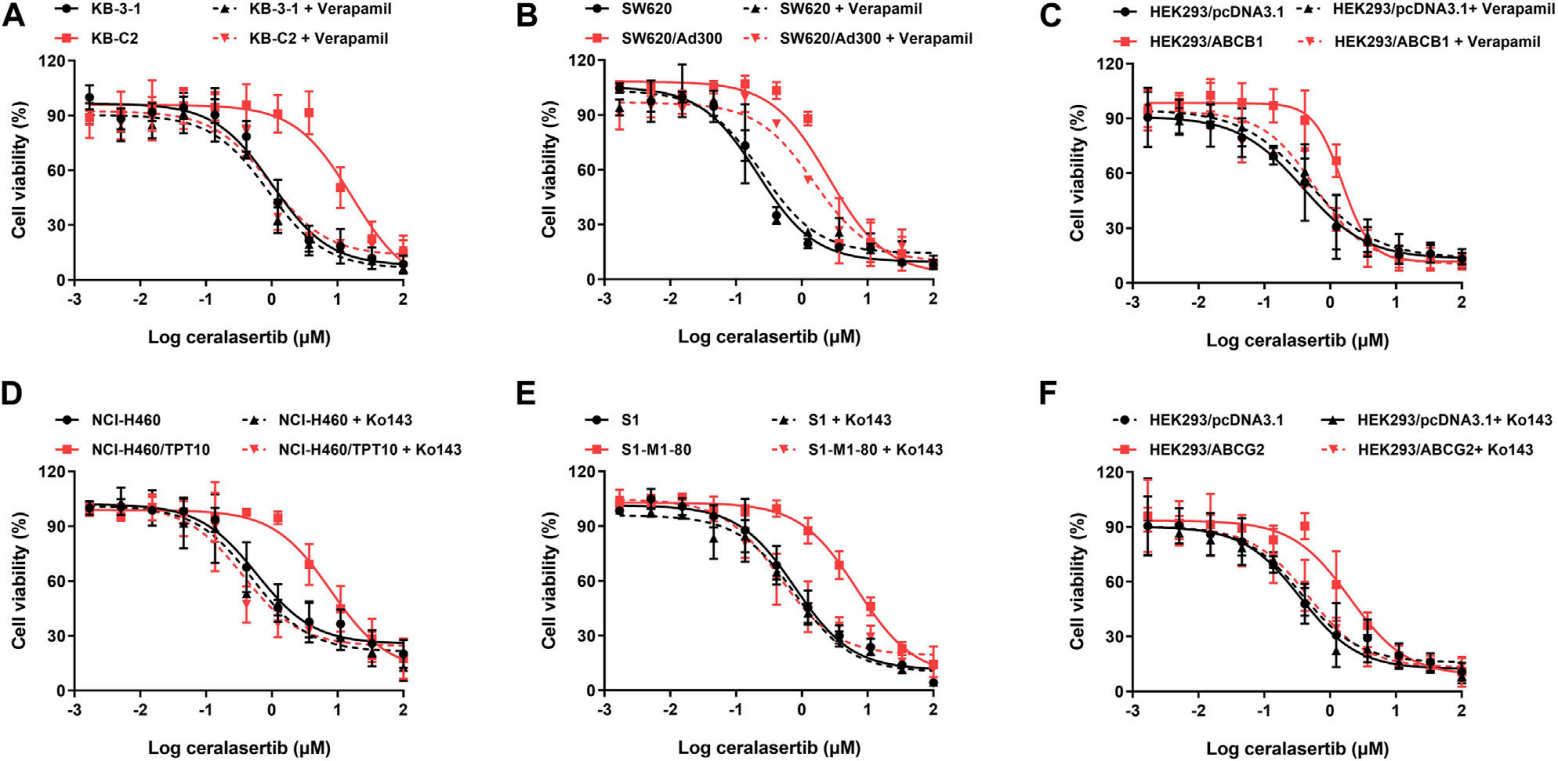
The cytotoxicity of ceralasertib in parental and drug resistant cells. Ceralasertib sensitivity is attenuated in P-gp-overexpressing cells KB-C2 **(A)**, SW620/Ad300 **(B)**, and HEK293/ABCB1 **(C)** as well as BCRP-overexpressing cells NCI-H460/TPT10 **(D)**, S1-M1-80 **(E)**, and HEK293/ABCG2 **(F)**. Data are expressed as mean ± SD from a representative of three independent experiments (*n* = 3).

To determine the efficacy of ceralasertib in P-gp or BCRP-overexpressing cells, we first performed the MTT assay to compare ceralasertib cytotoxicity in parental and drug-resistant cells. Overexpression of P-gp and BCRP were confirmed by Western blot as shown in Supplementary Figure S1. KB-C2 and SW620/Ad300 are selectively resistant to P-gp substrate such as colchicine and doxorubicin, while NCI-H460/TPT10 and S1-M1-80 cells are selectively resistant to BCRP substrate such as topotecan and mitoxantrone. The calculated IC_50_ and drug resistance fold are summarized in Tables 1, 2. As shown in Figures 1A, B, both P-gp-overexpressing drug-resistant cells KB-C2 and SW620/Ad300 were less sensitive to ceralasertib treatment compared to the parental cells KB-3-1 and SW620, as indicated by the 16-fold resistance in both pair of cells. Similarly, overexpression of BCRP decreased the sensitivity of ceralasertib in drug-resistant-cells NCI-H460/TPT10 and S1-M1-80 compared to that in the NCI-H460 and S1 cells (Figures 1D, E). Therefore, the cell viability data provides direct evidence that P-gp and BCRP can confer cancer cells resistance to ceralasertib. Furthermore, the results from gene transfected HEK293 cells were in line with cancer cell lines. In Figures 1C, F, both HEK293/ABCB1 and HEK293/ABCG2 cells were more resistant to ceralasertib than HEK293/pcDNA3.1 cells.

**TABLE 1:** Cytotoxicity of ceralasertib in parental and P-gp-overexpressing cells with or without P-gp inhibition.

Cell line	IC_50_ ± SD (μM)	Resistance fold
KB-3-1	1.01 ± 0.14	1
KB-C2	16.64 ± 3.70	16.41
KB-3-1 + Verapamil	0.89 ± 0.13	0.89
KB-C2 + Verapamil	0.96 ± 0.19	0.94
SW620	0.31 ± 0.26	1
SW620/Ad300	2.58 ± 1.48	8.39
SW620 + Verapamil	0.29 ± 0.01	0.93
SW620/Ad300+ Verapamil	1.29 ± 0.23	4.17
HEK293/pcDNA3.1	0.38 ± 0.13	1
HEK293/ABCB1	1.51 ± 0.39	3.95
HEK293/pcDNA3.1 + Verapamil	0.42 ± 0.18	1.11
HEK293/ABCB1+ Verapamil	0.52 ± 0.23	1.36

Verapamil is a selective inhibitor of P-gp transporter. IC50 values are represented as mean ± SD of at least three independent experiments performed in triplicate. Resistance fold was calculated by dividing the IC50 values of substrates in the presence or absence of inhibitor by the IC50 of parental cells without inhibitor.

**TABLE 2:** Cytotoxicity of ceralasertib in parental and BCRP-overexpressing cells with or without BCRP inhibition.

Cell line	IC_50_ ± SD (μM)	Resistance fold
NCI-H460	0.59 ± 0.09	1
NCI-H460/TPT10	7.86 ± 1.47	13.37
NCI-H460 + Ko143	0.51 ± 0.09	0.87
NCI-H460/TPT10 + Ko143	0.47 ± 0.08	0.80
S1	0.69 ± 0.27	1.00
S1-M1-80	6.82 ± 3.23	9.86
S1 + Ko143	0.70 ± 0.21	1.02
S1-M1-80 + Ko143	0.63 ± 0.31	0.91
HEK293/pcDNA3.1	0.33 ± 0.21	1
HEK293/ABCG2-WT	1.99 ± 0.24	6.00
HEK293/pcDNA3.1 + Ko143	0.27 ± 0.18	0.81
HEK293/ABCG2-WT+ Ko143	0.51 ± 0.13	1.52

Ko143 is a selective inhibitor of BCRP transporter. IC50 values are represented as mean ± SD of at least three independent experiments performed in triplicate. Resistance fold was calculated by dividing the IC50 values of substrates in the presence or absence of inhibitor by the IC50 of parental cells without inhibitor.

### 3.2 Inhibiting the efflux function of ABC transporters sensitized the MDR cells to ceralasertib treatment

The cell viability assay suggests that P-gp and BCRP may cause ceralasertib resistance in cancer cells. To confirm this finding, reference inhibitors verapamil and Ko143 were used to inhibit the function of P-gp and BCRP, respectively. As shown in Table 1, verapamil was able to decrease the resistance fold in drug resistant KB-C2 and SW620/Ad300 cells as well as gene transfected HEK293/ABCB1 cells. We observed complete reversal of ceralasertib resistance in KB-C2 cells (16.41-fold–0.94-fold) and partial reversal in SW620/AD300 cells (8.39-fold–4.17-fold), suggesting the involvement of multiple drug resistance mechanisms in SW620/AD300 cells. Table 2 shows that, in BCRP-overexpressing cells, Ko143 was able to significantly overcome ceralasertib resistance in both NCI-H460/TPT10 cells (13.37-fold–0.80-fold) and S1-M1-80 cells (9.86-fold–0.91-fold). Figure 1 also suggested that combining P-gp or BCRP inhibitors with ceralasertib can cause significant left shift of the cell viability curves in the drug-resistant cells. Therefore, the reversal studies indicated that P-gp and BCRP can regulate the sensitivity of ceralasertib in cancer cells.

### 3.3 The knockout of *ABCG2* gene restored the sensitivity to ceralasertib

**FIGURE 2:**
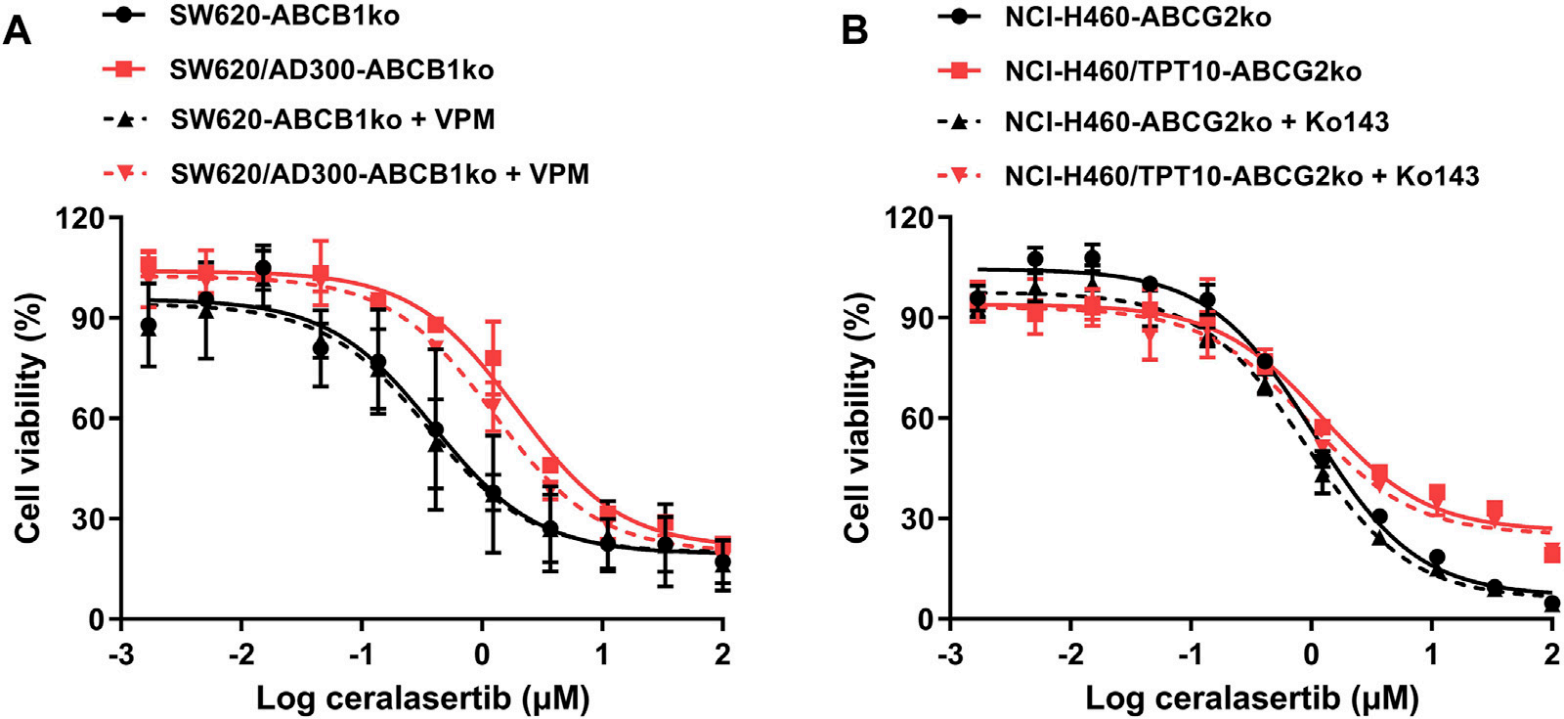
Knockout of P-gp and BCRP restored the sensitivity of ceralasertib in drug resistant cancer cells. The gene knockout cell lines were established by CRISPR-Cas9 system and used for ceralasertib sensitivity testing. **(A)** The cytotoxicity of ceralasertib in *abcb1* gene knockout cells with or without P-gp inhibitor verapamil. **(B)** The cytotoxicity of ceralasertib in *abcg2* gene knockout cells with or without BCRP inhibitor Ko143. Data are expressed as mean ± SD from a representative of three independent experiments (*n* = 3).

Drug-selected resistant cells may develop MDR through various mechanisms. Although we confirmed that overexpression of P-gp/BCRP is the major drug resistance mechanism in our drug-resistant cell lines, we cannot rule out the possibility that other mechanisms may contribute to ceralasertib resistance. In this case, *ABCB1* and *ABCG2* gene knockout models were used to further investigate the drug resistance mechanisms. As shown in Figure 2A, the drug resistance towards ceralasertib was partially reversed in SW620/Ad300 cells even with the knockout of *ABCB1* gene and the addition of ABCB1 inhibitor verapamil. Knockout of the *ABCB1* gene had minimal effect on the parental SW620 cells but decreased the resistance fold in the SW620/Ad300 cells (8.39-fold–5-fold). Although the IC_50_ summarized in Table 3 showed no significant difference, combined with our results in Table 2, this suggests that SW620/Ad300 cells processed multiple factors to induce ceralasertib resistance, which warrants further research to delineate the underlying mechanisms. In contrast, knock out of *ABCG2* gene was sufficient to mitigate ceralasertib resistance in NCI-H460/TPT10 cells (Figure 2B). Taken together, the data confirms that ceralasertib is a transported substrate of both P-gp and BCRP transporters, and that overexpression of P-gp/BCRP is sufficient to cause ceralasertib resistance in cancer cells.

**TABLE 3:** Cytotoxicity of ceralasertib in parental and ABCB1 or ABCG2 knockout cells with or without BCRP inhibition.

Cell line	IC_50_ ± SD (μM)	Resistance fold
SW620-ABCB1ko	0.39 ± 0.18	1
SW620/Ad300-ABCB1ko	1.95 ± 0.55	5.00
SW620-ABCB1ko + Verapamil	0.36 ± 0.21	0.92
SW620/Ad300-ABCB1ko + Verapamil	1.26 ± 0.16	3.23
NCI-H460-ABCG2ko	1.04 ± 0.09	1.00
NCI-H460/TPT10-ABCG2ko	1.24 ± 0.06	1.19
NCI-H460-ABCG2ko + Ko143	0.91 ± 0.14	0.86
NCI-H460/TPT10-ABCG2ko + Ko143	0.94 ± 0.12	0.90

Verapamil is a selective inhibitor of P-gp transporter and ko143 is a selective inhibitor of BCRP transporter. IC50 values are represented as mean ± SD of at least three independent experiments performed in triplicate. Resistance fold was calculated by dividing the IC50 values of substrates in the presence or absence of inhibitor by the IC50 of parental cells without inhibitor.

### 3.4 Ceralasertib did not affect the biological functions of BCRP but downregulated the expression of P-gp

**FIGURE 3:**
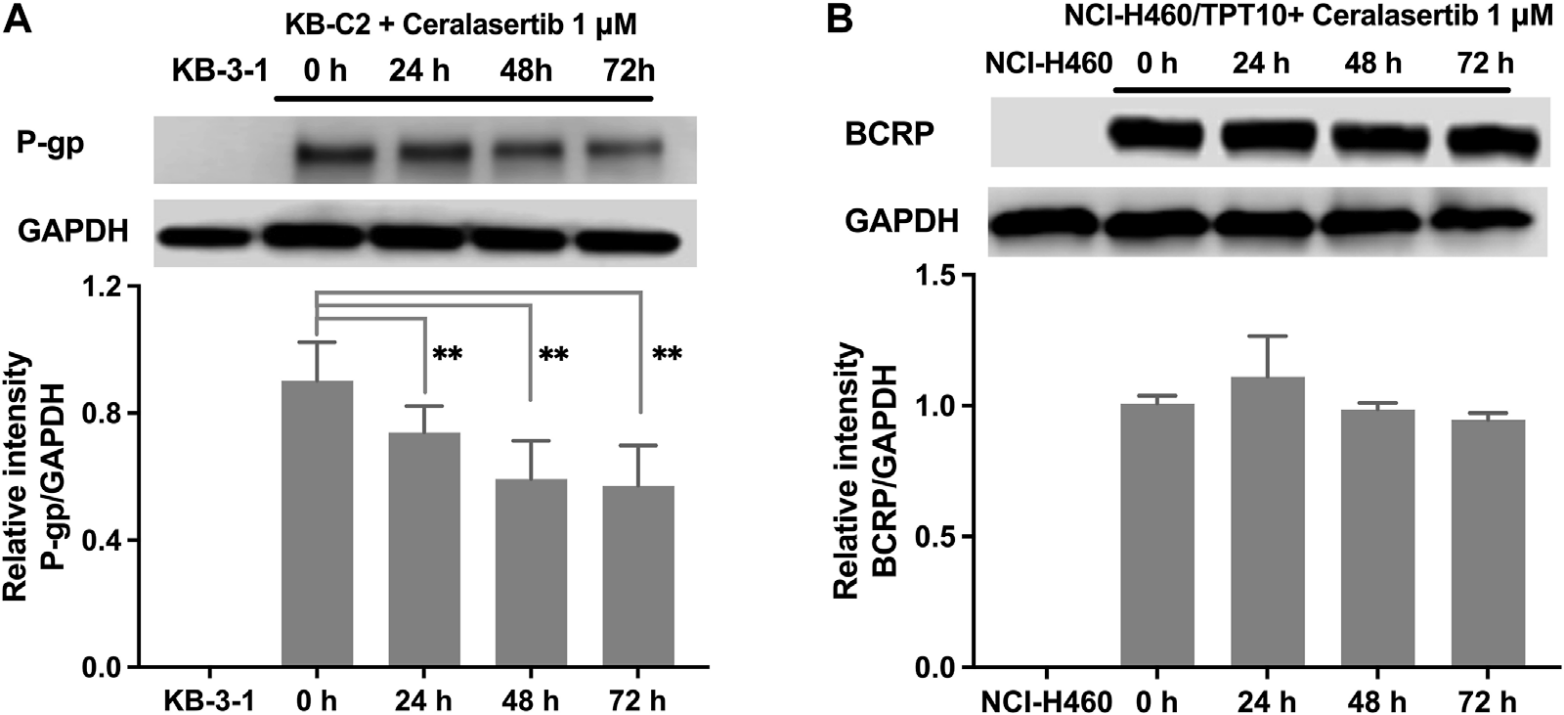
The effect of ceralasertib on P-gp and BCRP protein expression level. **(A)** The time-dependent effect of ceralasertib on the expression levels of P-gp in KB-C2 cells. **(B)** The time-dependent effect of ceralasertib on the expression levels of BCRP in NCI-H460/TPT10 cells. Data are expressed as mean ± SD from three independent experiments (*n* = 3). ∗*p* < 0.05 *versus* the corresponding control group.

**FIGURE 4:**
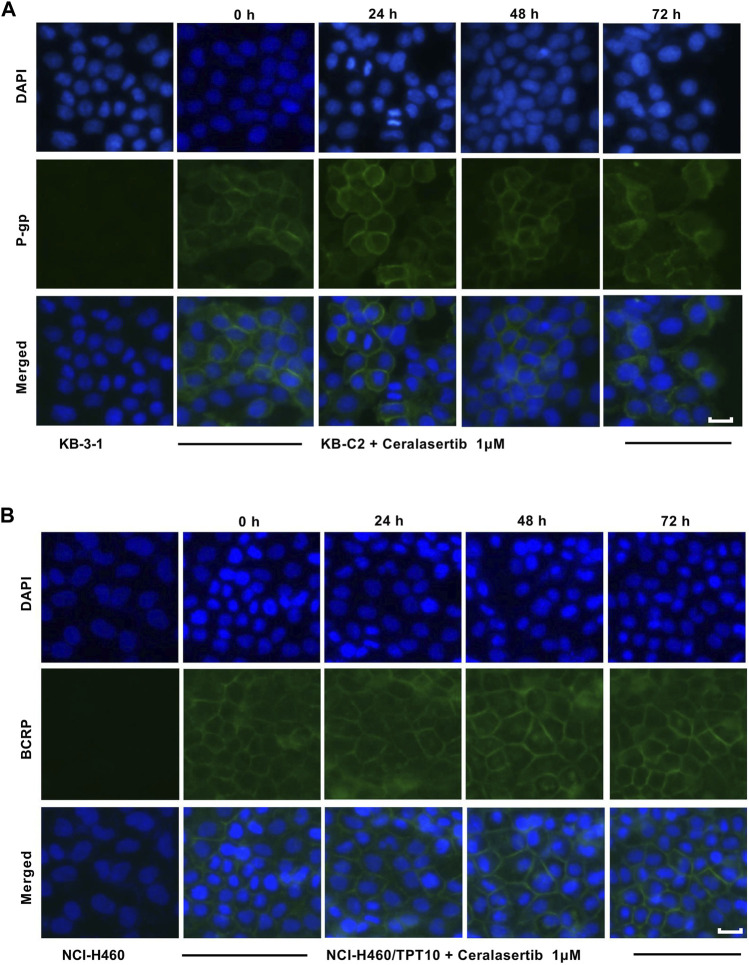
The effect of ceralasertib on ABC transporters cell surface localization. **(A)** Cell membrane localization of P-gp in drug resistant KB-C2 cells incubated with 1 μM of ceralasertib for up to 72 h. **(B)** Cell membrane localization of BCRP in drug resistant NCI-H460/TPT10 cells incubated with 1 μM of ceralasertib for up to 72 h. Scale bar 10 μm.

**FIGURE 5:**
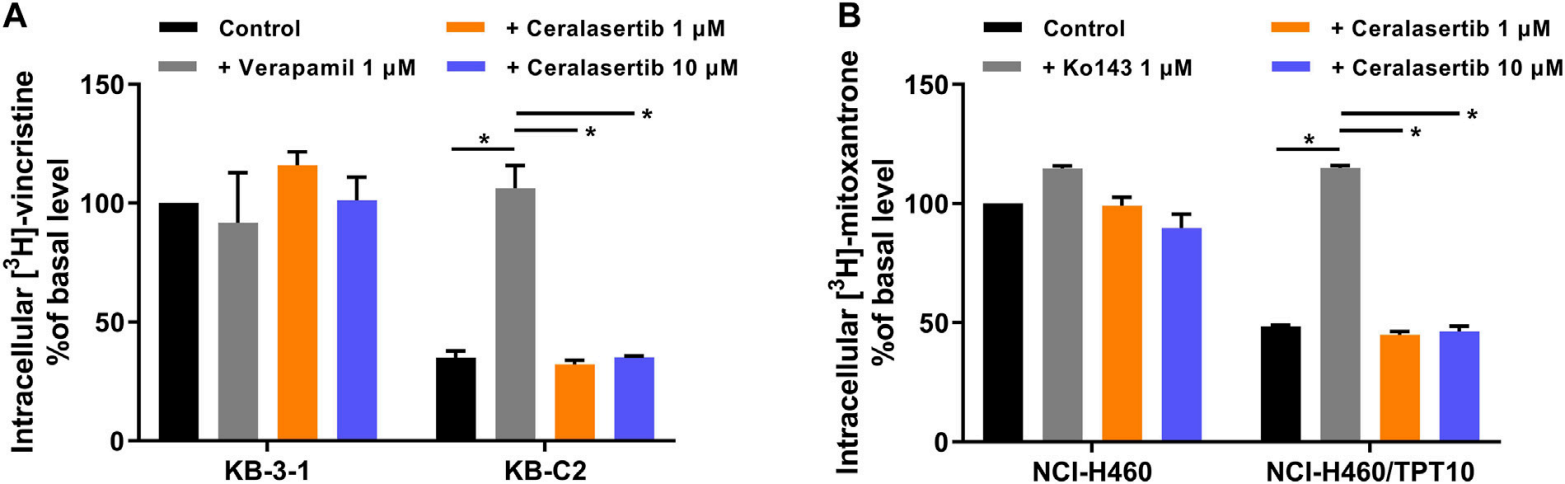
The effect of ceralasertib on the substrate efflux function of P-gp and BCRP transporters. **(A)** P-gp-overexpressing KB-C2 cells have decreased intracellular level of [^3^H]-vincristine, which can be reversed by verapamil but not ceralasertib. **(B)** BCRP-overexpressing NCI-H460/TPT10 cells have decreased intracellular level of [^3^H]-mitoxantrone, which can be reversed by Ko143 but not ceralasertib. Data are expressed as mean ± SD from three independent experiments (*n* = 3). ∗*p* < 0.05 *versus* the corresponding control group.

To further characterize the impact of ceralasertib on P-gp/BCRP protein expression, Western blot and immunofluorescent assay were performed. Protein expression levels after ceralasertib treatment were determined by Western blot as shown in Figure 3. At low toxic concentration, ceralasertib did not induce or downregulate the protein levels of BCRP in BCRP-overexpressing NCI-H460/TPT10 cells. However, ceralasertib time-dependently downregulated the expression of P-gp in P-gp-overexpressing KB-C2 cells. In addition, ceralasertib at 1 μM had no effect on the localization of P-gp and BCRP (Figure 4). Both transporters are expressed on cell surface without internalization after treatment. Furthermore, the most significant function of ABC transporters is the substrate efflux ability. We utilized tritium-labeled substrate to quantify the efflux activity under ceralasertib treatment. As shown in Figure 5A, the P-gp overexpressing cancer cells KB-C2 had significantly lower accumulation of P-gp substrate vincristine than the parental cells KB-3-1. The P-gp inhibitor verapamil was able to inhibit P-gp efflux function and restore the accumulation level of vincristine in KB-C2 cells to that of the KB-3-1 cells. Compared to verapamil, ceralasertib at both low concentration 1 μM and high concentration 10 μM did not alter the intracellular level of vincristine in KB-C2 cells. We observed a similar trend in BCRP-overexpressing NCI-H460/TPT10 cells. While the BCRP inhibitor Ko143 was able to increase the retention of BCRP substrate mitoxantrone in the drug resistant cells, ceralasertib failed to produce any effect (Figure 5B). Combined, these assays suggest that ceralasertib is unlikely to affect the biological functions of the two ABC transporters.

### 3.5 Ceralasertib stimulated the P-gp and BCRP ATPases in crude membrane fraction

**FIGURE 6:**
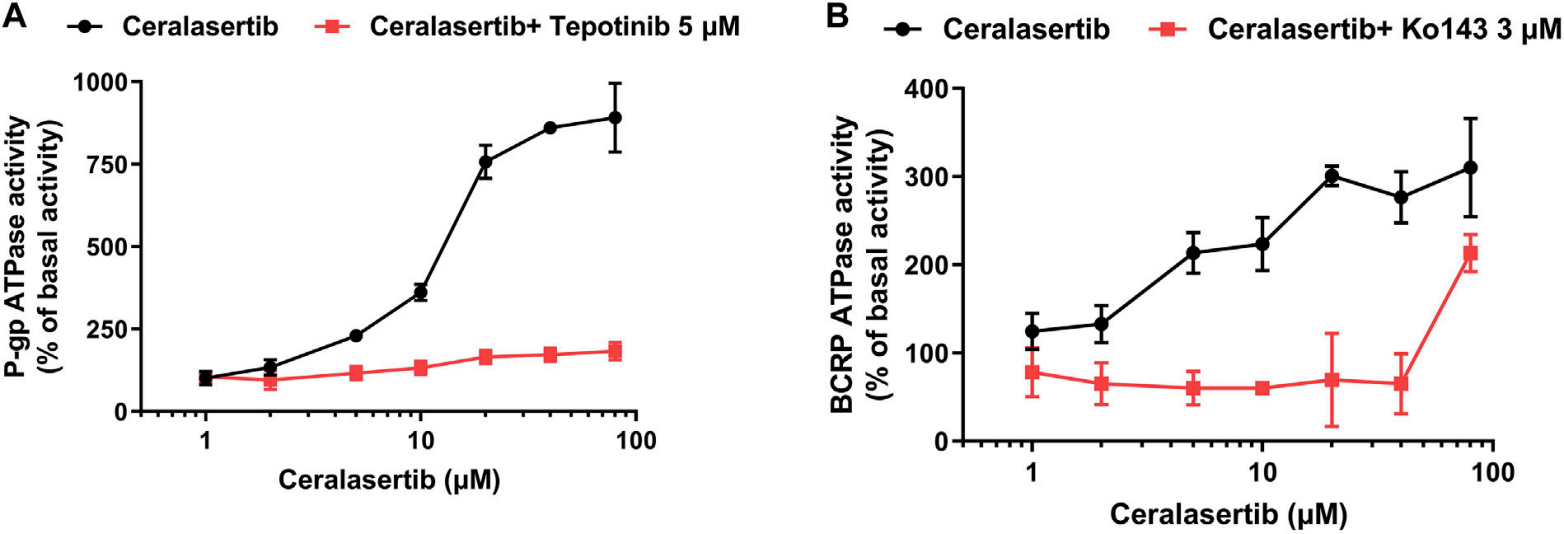
Ceralasertib stimulated the ATPase activities of both P-gp and BCRP transporters. **(A)** Ceralasertib at 0–80 μM stimulated P-gp ATPase function, which can be blocked by the selective P-gp ATPase inhibitor tepotinib. **(B)** Ceralasertib at 0–80 μM upregulated BCRP ATPase function, which can be mitigated by the selective BCRP ATPase inhibitor Ko143. Data are expressed as mean ± SD from three independent experiments (*n* = 3).

ABC transporters use the energy derived from ATP hydrolysis to drive the transportation of its substrates. Therefore, an ATPase assay was performed to confirm the activation of P-gp and BCRP ATPase when treated with ceralasertib. As shown in Figure 6, ceralasertib concentration-dependently stimulated the function of P-gp ATPase to a maximal of 8.9-fold (Figure 6A) and BCRP ATPase to a maximal of 3.1-fold (Figure 6B). To further validate that the increased ATP hydrolysis is indeed caused by P-gp/BCRP ATPase, we used the reference inhibitors for both ATPase. Tepotinib, a P-gp ATPase inhibitor, was able to significantly block the activation caused by ceralasertib. And the selective BCRP ATPase inhibitor Ko143 was able to completely mitigate ceralasertib-induced ATP hydrolysis at 0–40 μM. Ko143 was not effective when 80 μM of ceralasertib was used, which could be due to the competition of drug-binding site. The selective inhibitors were able to suppress the ATPase activity compared to baseline level as shown in Supplementary Figure S2. The ATPase assay suggests that ceralasertib can stimulate P-gp/BCRP ATPase function and confirm its role as substrate of P-gp/BCRP.

### 3.6 Ceralasertib docked into P-gp and BCRP substrate-binding site with high affinities

**FIGURE 7:**
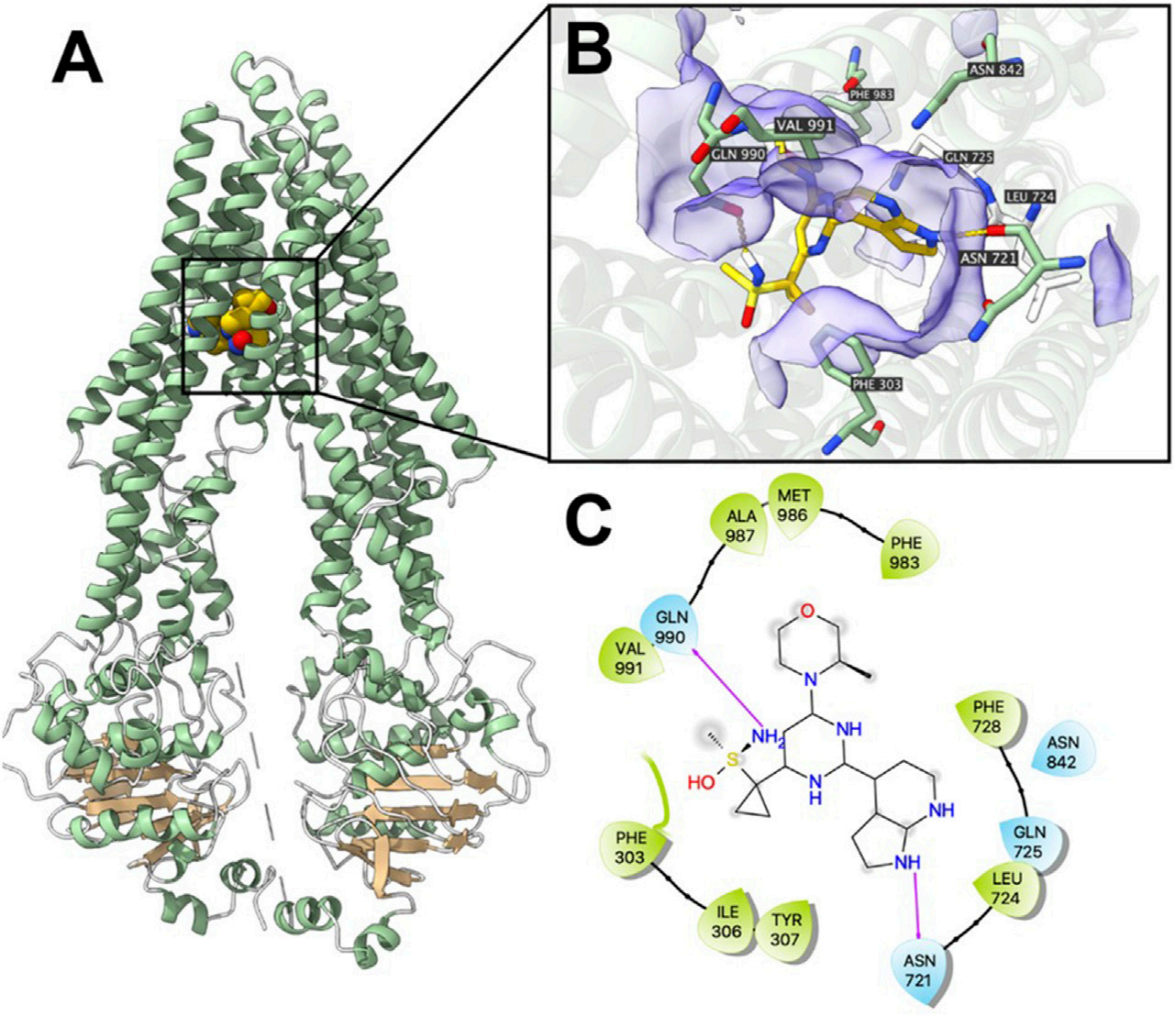
Computational docking analysis of the highest-scoring docked pose of ceralasertib within human P-gp protein model at substrate-binding site. **(A)** Overview of paclitaxel and the best-scoring pose of ceralasertib in the drug binding pocket of P-gp protein. **(B)** Details of interactions between ceralasertib and P-gp binding pocket. Predicted hydrogen bonds were displayed as yellow dash lines. **(C)** Two-dimension ceralasertib—P-gp interaction. Important amino acids were displayed as colored bubbles (green: hydrophobic; blue: polar). Predicted hydrogen bonds were displayed as purple lines with arrows.

**FIGURE 8:**
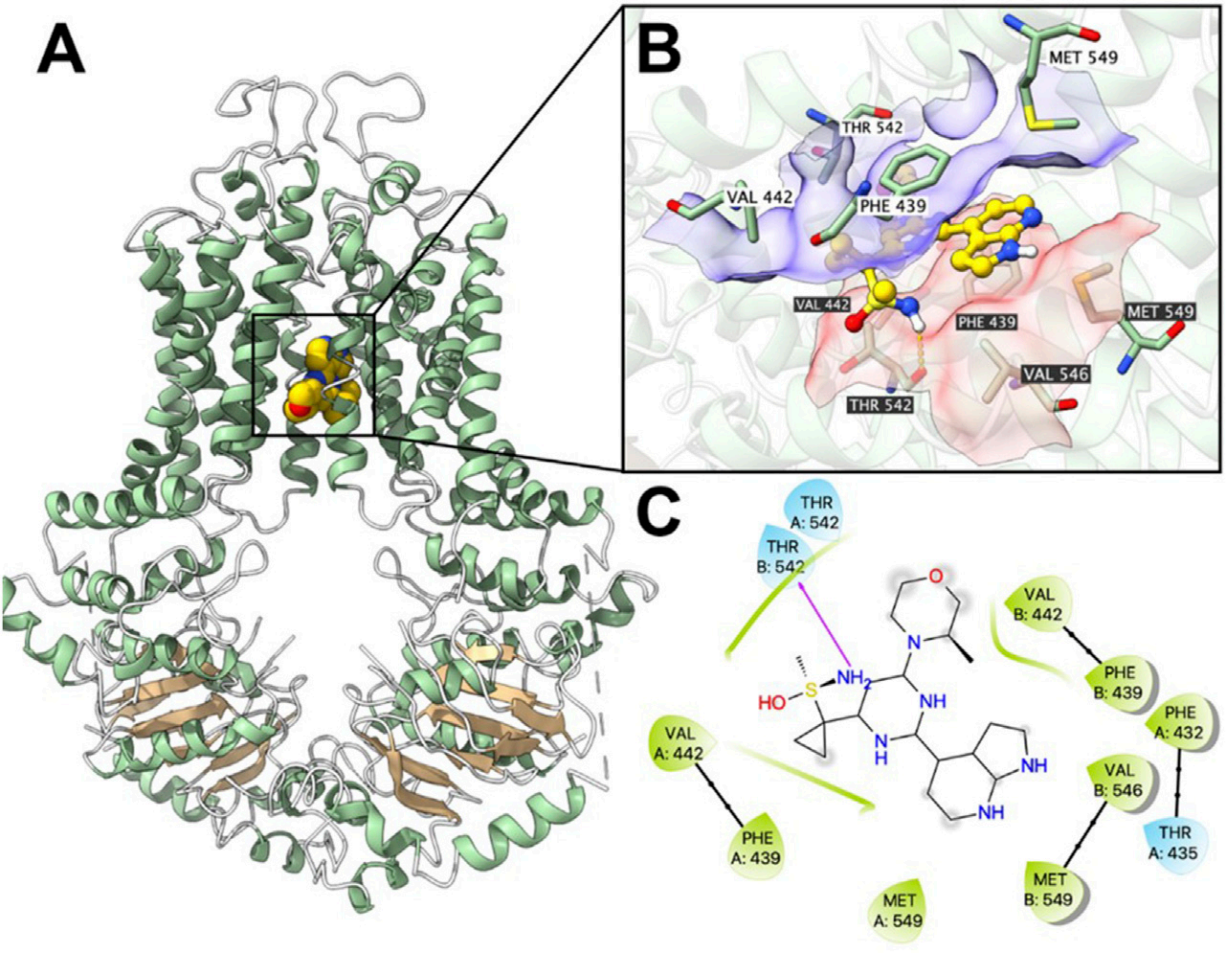
Computational docking analysis of the highest-scoring docked pose of ceralasertib within human BCRP protein model at substrate-binding site. **(A)** Overview of the best-scoring pose of ceralasertib in the drug binding pocket of BCRP protein. **(B)** Details of interactions between ceralasertib and BCRP binding pocket. Predicted bonds were displayed as colored dash lines: hydrogen bond: yellow. Labels with white or black background indicate chain A or B, respectively. **(C)** Two-dimension ceralasertib—BCRP interaction. Important amino acids were displayed as colored bubbles (green: hydrophobic; blue: polar). Predicted hydrogen bond was displayed as purple lines with arrows.

Computational docking analysis was performed to predict the binding interaction between ceralasertib and both ABC transporters. The results showed that ceralasertib docked into the P-gp substrate binding site with an affinity score of −7.7 kcal/mol. Details of ligand-receptor interaction were displayed in Figure 7. Ceralasertib is positioned and stabilized in the hydrophobic cavity formed by Phe303, Ile306, Tyr307, Leu724, Phe728, Phe983, Met986, Ala987 and Val991. Additionally, ceralasertib was stabilized by hydrogen bonds formed with Asn721 and Gln990. Furthermore, the results showed that ceralasertib docked into the BCRP substrate binding site with an affinity score of −9.6 kcal/mol. Details of ligand-receptor interaction were displayed in Figure 8. Ceralasertib is positioned and stabilized in the hydrophobic cavity formed by Phe432, Phe439, Val442, Met549 (chain A) and Val442, Phe439, Val546, Met549 (chain B). Additionally, ceralasertib was stabilized by hydrogen bond formed with Thr542 (Chain B).

## 4 Discussion

The DDR pathway consists of complex protein interactions to maintain cell viability and prevent neoplasia by regulating the cell cycle, apoptosis, metabolism, chromatin remodeling, and immunogenicity (Pilie et al., 2019). While faults in DDR are known as drivers and hallmarks of cancer, it provides the rationale of DDR inhibition-based cancer therapy (Cheng et al., 2022). ATR kinase plays a key role in recognizing and inducing a response to replication stress (Saldivar et al., 2017). Increased replication stress, caused by oncogene activation or DNA-damaging agents, could lead to DNA breaks that are highly toxic to cancer cells (Gaillard et al., 2015). Thus, inhibition of ATR kinase activity will suppress the normal response to replication stress and can be utilized for cancer therapy. Preclinical studies and early-phase clinical trials have demonstrated that ATRi can induce antitumor effects without severe toxicity (Yano and Shiotani, 2023). Multiple ATRi are currently under clinical evaluation for solid tumors, such as elimusertib, berzosertib, and gartisertib. Ceralasertib, developed by AstraZeneca, is being investigated as monotherapy and combinational therapy in solid tumors and hematological malignancy. In clinical settings, an important factor that determines the efficacy of ATRi is the development of drug resistance. Therefore, understanding the underlying mechanisms is critical for achieving optimal therapeutic efficacy and developing strategies to overcome drug resistance.

Since P-gp and BCRP are well known ABC transporters that contribute to MDR in cancer, we tested the efficacy of ceralasertib in four sets of drug resistant cell models. Colchicine-selected KB-C2 and doxorubicin-selected SW620/Ad300 cells overexpress P-gp protein compared to the parental KB-3-1 and SW620 cells. Topotecan-selected NCI-H460/TPT10 and mitoxantrone-selected S1-M1-80 cells are BCRP-overexpressing cells established from the parental NCI-H460 and S1 cells. The cell viability assay reveals that P-gp and BCRP overexpression can render cancer cells resistance to ceralasertib. Furthermore, the drug resistance can be reversed by combining ceralasertib with P-gp or BCRP inhibitors. Because drug-selected cell line may exhibit MDR phenotype by multiple drug resistance mechanisms (Bukowski et al., 2020), cell viability assay was performed in gene transfected HEK293 cells and gene knockout cancer cells to further evaluate the relationship between ceralasertib and P-gp/BCRP. The results show that HEK293 cells transfected with *ABCB1* or *ABCG2* gene were resistant to ceralasertib, which can be completely mitigated by P-gp inhibitor verapamil or BCRP inhibitor Ko143. When *ABCG2* gene was knockout from the drug resistant NCI-H460/TPT10 cells, the cells restored the sensitivity to ceralasertib. Interestingly, knockout of *ABCB1* gene did not achieve complete reversal effect in SW620/Ad300 cells, suggesting the existence of another drug resistance mechanism. This effect agrees with our observations that verapamil can only reverse ceralasertib resistance from 8.39- to 4.17-fold in SW620/Ad300 cells. Further investigation on the doxorubicin-selected SW620/Ad300 cells may provide more insight into other drug resistance mechanisms of ceralasertib. To summarize, we confirm that overexpression of P-gp and BCRP is sufficient to cause drug resistance to ceralasertib treatment.

ABC transporters are known for their ability to translocate substrates across cell membranes, which is driven by ATP hydrolysis (Flatt et al., 2023). The ATPase assay demonstrates that, in the presence of ceralasertib, the ATPase function of P-gp and BCRP are significantly stimulated from the basal level as measured by ATP hydrolysis. Furthermore, ceralasertib-induced ATP hydrolysis was inhibited with the use of tepotinib and Ko143, which specifically inhibit the activity of P-gp and BCRP ATPases. This data provides direct evidence that ceralasertib is a substrate of P-gp and BCRP, therefore susceptible to P-gp- and BCRP-mediated MDR. The docking analysis simulated a high affinity for the binding of ceralasertib with the substrate binding sites of P-gp and BCRP. For both transporters, ceralasertib is predicted to be positioned and stabilized in the hydrophobic cavity formed with multiple surrounding amino acids. Although the computational analysis does not reveal the true binding pose, it could serve as a reference for the drug-transporter interactions.

It has been reported that some tyrosine kinase inhibitors can regulate the function of ABC transporters by manipulating the protein expression (Nakanishi et al., 2006; Pick and Wiese, 2012; Sen et al., 2012). To this end, we performed Western blot and immunofluorescence staining to investigate this possibility. After 72 h of incubation with ceralasertib, immunostaining confirms that P-gp and BCRP are only expressed on cell membranes. Besides, ceralasertib did not induce significant changes in BCRP expression. In contrast, low concentration ceralasertib treatment was able to decrease P-gp level in a time-dependent manner. The decrease of P-gp may alter the efficacy of ceralasertib during long term treatment since it is characterized as a P-gp substrate. However, a potential application of ATRi would be repurposing them to antagonize P-gp-mediated MDR by downregulating the protein expression in drug resistant tumors. To our knowledge, this is the first report to show that ATR inhibitor can regulate P-gp expression level. Further studies are needed to reveal the interaction of ATR signaling with P-gp.

Substrates such as GSK1070916 (Wu et al., 2021), palbociclib (Fu et al., 2022), and tivantinib (Wu et al., 2020) are shown to competitively inhibit the transportation of another substrate in presence, which cause drug-drug interactions at transporter level. The tritium-labeled drug accumulation assay ruled out this possibility as the drug resistant cells maintained minimum substrate accumulation even with high concentrations of ceralasertib. In contrast, P-gp inhibitor verapamil and BCRP inhibitor Ko143 were able to increase substrate accumulation to a similar level as parental cells (Liu et al., 2022; Teng et al., 2024). Therefore, the results suggest that ceralasertib is unlikely to modulate the biological functions of P-gp and BCRP during short-term exposure. However, whether ceralasertib could induce or downregulate the expression of P-gp and BCRP after long-term treatment requires further study.

In conclusion, we describe for the first time that the cytotoxicity of ceralasertib in cancer cells is modulated by P-gp and BCRP expression level. As many ATRi share similarities in their chemical structure, it is possible that P-gp- and BCRP-mediated MDR can be a common drug resistance mechanism. Future work should focus on the significance of P-gp and BCRP in mediating ceralasertib efficacy in comprehensive preclinical models and clinical settings.

## Data Availability

The original contributions presented in the study are included in the article/Supplementary Material, further inquiries can be directed to the corresponding authors.
