# Supplementary material for: Multidrug resistance transporters P-gp and BCRP limit the efficacy of ATR inhibitor ceralasertib in cancer cells
Source: Front Pharmacol. 2024 May 2;15:1400699. doi: 10.3389/fphar.2024.1400699 (PMC11096521; doi:10.3389/fphar.2024.1400699)
Supplement: Supplementary file 1 [file DataSheet1.DOCX]

Supplementary Material

Multidrug resistance transporters P-gp and BCRP limit the efficacy of ATR inhibitor ceralasertib in cancer cells.

**Xuan-Yu Chen^1,2 #^, Zhuo-Xun Wu^2, #^, Jing-Quan Wang^2^, Qiu-Xu Teng^2^, Hailin Tang^3^,** **Qianwen Liu^3,^*, Zhe-Sheng Chen^1,2^*, Wenkuan Chen^3,^***

*** Correspondence:** Qianwen Liu: liuqianw@sysucc.org.cn, Zhe-Sheng Chen: chenz@stjohns.edu, Wenkuan Chen: chenwk@sysucc.org.cn.


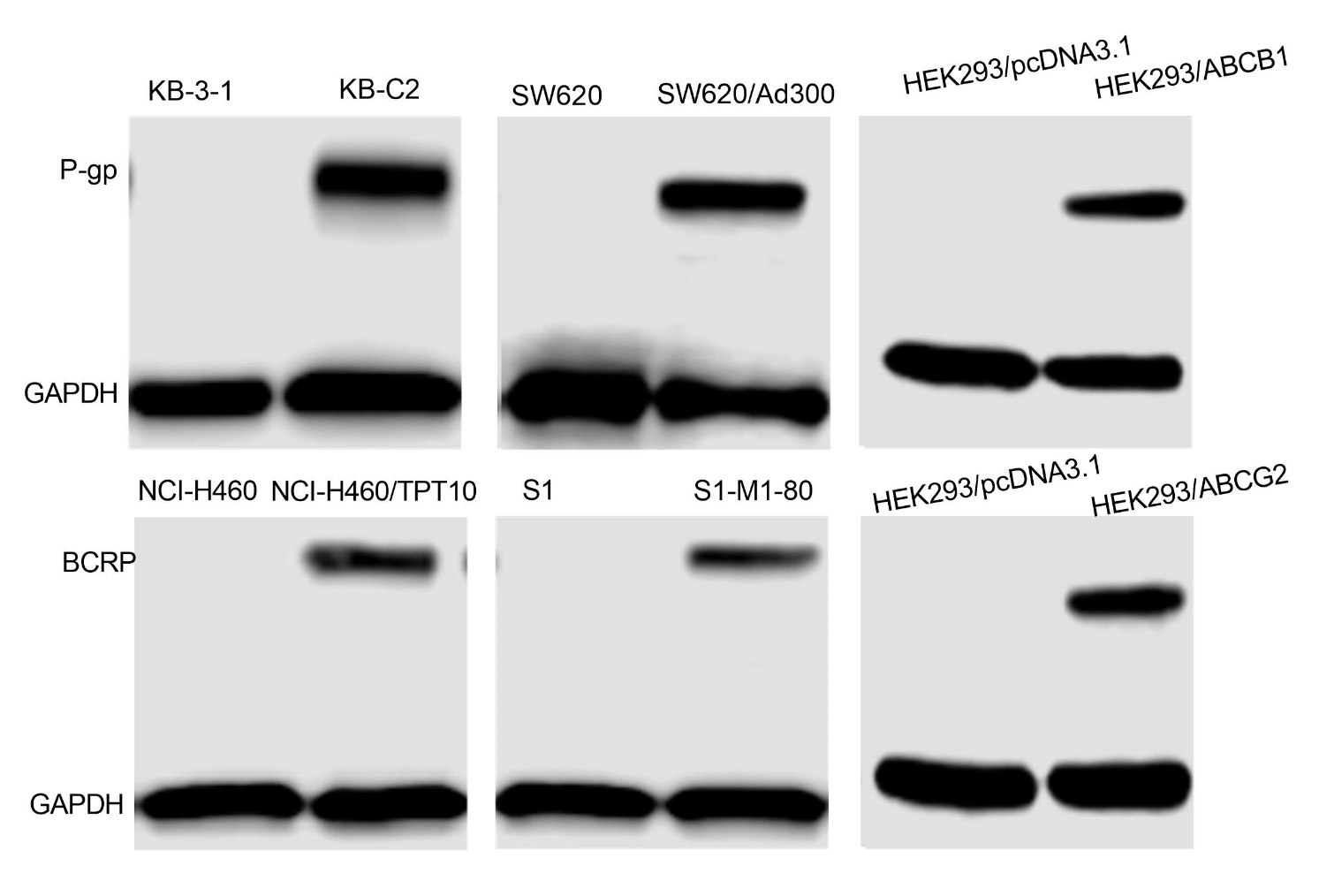


**Supplementary Figure 1. Protein expression of P-gp and BCRP in parental and drug resistant cells.** KB-3-1, SW620, and HEK293/pcDNA3.1 are parental cells that do not express P-gp protein. KB-C2, SW620/Ad300, and HEK293/ABCB1 are drug resistant cells that overexpress P-gp Protein. NCI-H460, S1, and HEK293/pcDNA3.1 are parental cells that do not express BCRP protein. NCI-H460/TPT10, S1-M1-80, and HEK293/ABCG2 are drug resistant cells that overexpress BCRP protein.


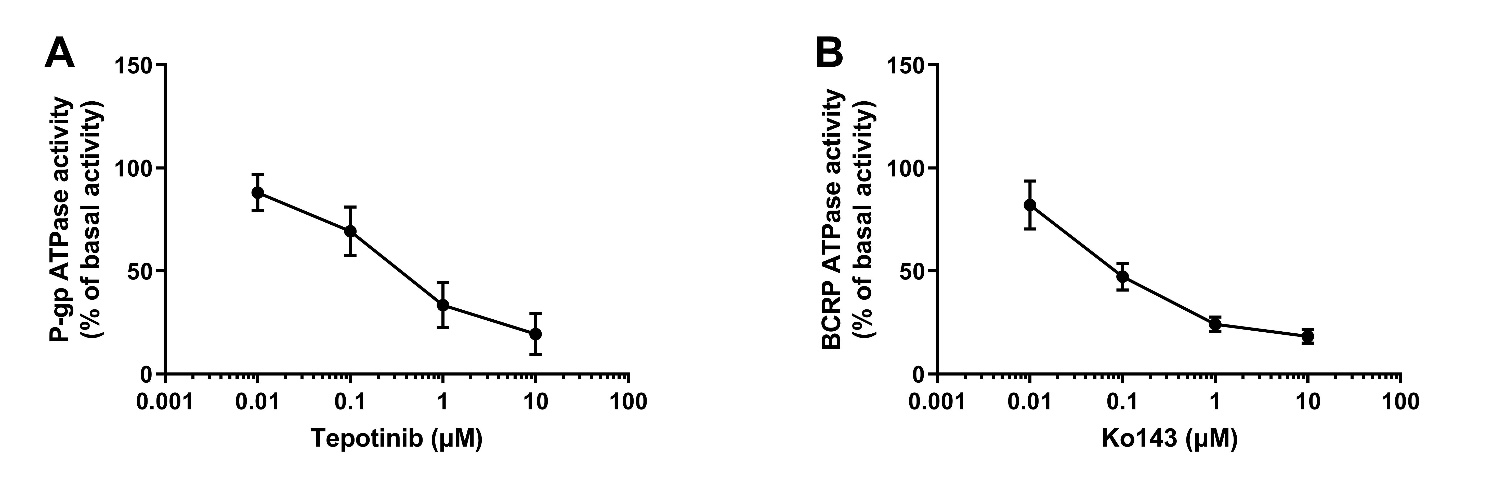


**Supplementary Figure 2. Inhibition of ATPase activity by tepotinib and Ko143.** (A) Selective P-gp ATPase inhibitor tepotinib at 0-10 μM inhibited P-gp ATPase function. (B) selective BCRP ATPase inhibitor Ko143 at 0-10 μM inhibited BCRP ATPase function. Data are expressed as mean ± SD from three independent experiments (*n* = 3).
